# Dopamine Modulates Adaptive Forgetting in Medial Prefrontal Cortex

**DOI:** 10.1523/JNEUROSCI.0740-21.2022

**Published:** 2022-08-24

**Authors:** Francisco Tomás Gallo, María Belén Zanoni Saad, Azul Silva, Juan Facundo Morici, Magdalena Miranda, Michael C. Anderson, Noelia V. Weisstaub, Pedro Bekinschtein

**Affiliations:** ^1^Laboratorio de Memoria y Cognición Molecular, Instituto de Neurociencia Cognitiva y Traslacional, Consejo Nacional de Investigaciones Científicas y Técnicas(CONICET)-Fundación INECO-Universidad Favaloro, 1071 Ciudad Autónoma de Buenos Aires, Argentina; ^2^Grupo de Neurociencia de Sistemas, Instituto de Fisiología y Biofísica “Houssay” (IFIBIO “Houssay”), Universidad de Buenos Aires, Consejo Nacional de Investigaciones Científicas y Técnicas (CONICET), C1121ABG Ciudad Autónoma de Buenos Aires, Argentina; ^3^MRC Cognition and Brain Sciences Unit, University of Cambridge, Cambridge CB2 7EF, United Kingdom; ^4^Behavioural and Clinical Neurosciences Unit, University of Cambridge, Cambridge CB2 3EB, United Kingdom

**Keywords:** active forgetting, D_1_ receptors, inhibitory control, medial prefrontal cortex, retrieval-induced forgetting

## Abstract

Active forgetting occurs in many species, but how behavioral control mechanisms influence which memories are forgotten remains unknown. We previously found that when rats need to retrieve a memory to guide exploration, it reduces later retention of other competing memories encoded in that environment. As with humans, this retrieval-induced forgetting relies on prefrontal control processes. Dopaminergic input to the prefrontal cortex is important for executive functions and cognitive flexibility. We found that, in a similar way, retrieval-induced forgetting of competing memories in male rats requires prefrontal dopamine signaling through D_1_ receptors. Blockade of medial prefrontal cortex D_1_ receptors as animals encountered a familiar object impaired active forgetting of competing object memories as measured on a later long-term memory test. Inactivation of the ventral tegmental area produced the same pattern of behavior, a pattern that could be reversed by concomitant activation of prefrontal D_1_ receptors. We observed a bidirectional modulation of retrieval-induced forgetting by agonists and antagonists of D_1_ receptors in the medial prefrontal cortex. These findings establish the essential role of prefrontal dopamine in the active forgetting of competing memories, contributing to the shaping of retention in response to the behavioral goals of an organism.

**SIGNIFICANCE STATEMENT** Forgetting is a ubiquitous phenomenon that is actively promoted in many species. The very act of remembering some experiences can cause forgetting of others, in both humans and rats. This retrieval-induced forgetting process is thought to be driven by inhibitory control signals from the prefrontal cortex that target areas where the memories are stored. Here we started disentangling the neurochemical signals in the prefrontal cortex that are essential to retrieval-induced forgetting. We found that, in rats, the release of dopamine in this area, acting through D_1_ receptors, was essential to causing active forgetting of competing memories. Inhibition of D_1_ receptors impaired forgetting, while activation increased forgetting. These findings are important, because the mechanisms of active forgetting and their linkage to goal-directed behavior are only beginning to be understood.

## Introduction

Neuroscientific accounts of forgetting often have focused on the passive decay of memory traces (Davis and Zhong, 2017). However, recent neurobiological studies indicate that active forgetting mechanisms also can dictate the fate of a memory (Berry et al., 2012; Akers et al., 2014; Liu et al., 2016; Migues et al., 2016; Davis and Zhong, 2017; Awasthi et al., 2019). A common feature of both active forgetting processes and passive decay is that they are indifferent to memory content, but there is the question of whether the forgetting of particular traces may be adaptively prioritized to benefit the goals of the organism. Selective forgetting mechanisms have been described that adaptively tune the accessibility of memories to the behavioral demands of the organisms (Anderson, 2003; Anderson and Hulbert, 2021). When people and rats retrieve a past event, other memories that compete with and hinder retrieval are more likely to be forgotten (Anderson et al., 1994; Bekinschtein et al., 2018). This “retrieval-induced forgetting” (RIF) occurs for a broad range of stimuli and contexts (Anderson and Hulbert, 2021; Anderson and Marsh, 2021). In humans, retrieval-induced forgetting arises because trying to retrieve a specific memory triggers an inhibitory control mechanism mediated by the lateral prefrontal cortex that focuses retrieval on goal-relevant traces by suppressing distracting memories (Anderson and Spellman, 1995; Anderson, 2003). Paralleling these findings, rats can also engage this active forgetting mechanism to inhibit competing memories. As in humans, RIF in rats requires prefrontal engagement during the selective retrieval practice (RP) phase (Wu et al., 2014; Bekinschtein et al., 2018), and yields long-lasting forgetting that generalizes across multiple retrieval cues (Bekinschtein et al., 2018). In mammals, the prefrontal cortex facilitates flexible behavior (Miller and Cohen, 2001; Dalley et al., 2004; Ragozzino, 2007; Aron et al., 2014) via control mechanisms that suppress habitual responses that might otherwise dominate goal-directed action and have also been associated with attentional processes (Dalley et al., 2004; Aron et al., 2014; Wessel and Aron, 2017). In rodents, the medial prefrontal cortex (mPFC) has been associated with attentional and inhibitory control processes (Miller and Cohen, 2001; Dalley et al., 2004; Ragozzino, 2007; Friedman and Robbins, 2022). We have proposed that the mPFC also suppresses competing memories, initiating a key signal that triggers active forgetting (Bekinschtein et al., 2018).

Dopamine is essential for cognitive control mechanisms mediated by the prefrontal cortex of humans, monkeys, and rodents (Friedman and Robbins, 2022). In the mPFC, dopamine modulates processes such as working memory (Sawaguchi and Goldman-Rakic, 1991; Zahrt et al., 1997; Granon et al., 2000; Vijayraghavan et al., 2007), attention, and behavioral flexibility (Ragozzino, 2002; Floresco, 2013). The rodent mPFC receives a dopaminergic input from neurons in the ventral tegmental area (VTA) that innervates both pyramidal cells and interneurons. In particular, D_1_ dopamine receptors (D_1_Rs) in the mPFC are critical for mediating dopamine effects on cognitive functioning (Floresco et al., 2006). Interestingly, an imaging genetics study in humans has linked genetic variation in prefrontal dopamine levels to differences in the engagement of lateral prefrontal cortex during selective retrieval and, correspondingly, to adaptive forgetting (Wimber et al., 2011). Here, we investigated whether dopamine-mediated control processes in the mPFC contribute to adaptive forgetting of competing memories in our rodent model of retrieval-induced forgetting. We found that the blockade of D_1_Rs in mPFC of rats abolished retrieval-induced forgetting of object memories. To strengthen our hypothesis that control processes are involved in retrieval-induced forgetting, we also showed that the same manipulation of the D_1_Rs prevented animals from switching to a new rule in a set-shifting task that involves control processes to change a course of action (Ragozzino et al., 1999; Floresco et al., 2006). Inactivating VTA activity also impaired forgetting, and we could reverse this impairment by concurrently activating D_1_Rs in mPFC. Importantly, we show that dopaminergic modulation of adaptive forgetting is bidirectional, as activation of D_1_R in mPFC significantly enhances retrieval-induced forgetting. Our results suggest that dopamine-dependent mechanisms of cognitive control over memory are conserved across species and are essential for adaptive forgetting in the mammalian brain.

## Materials and Methods

### Ethics statement

All experimental procedures were conducted in accordance with institutional regulations (Institutional Animal Care and Use Committee of the School of Medicine, University of Buenos Aires, ASP #49527/15) and government regulations (SENASAARS617.2002). All efforts were made to minimize the number of animals used and their suffering.

### Subjects

Two hundred forty-five male adult Wistar rats (weight range, 180–250 g) were housed up to five per cage and were kept with water and food available *ad libitum* under a 12 h light/dark cycle (lights on at 7:00 A.M.) at a constant temperature range of 21–23°C. Separate groups of animals were used for the different experiments. Experiments took place during the light phase of the cycle. The experimental protocol for this study followed guidelines of the National Institutes of Health in the *Guide for the Care and Use of Laboratory Animals*. The number of animals used is stated for each experiment (see below).

### Apparatus

Different arena contexts were used during the experiments.

The design for most of the experiments was mixed factorial designs with a within-subjects manipulation of drug versus vehicle (Veh) and a between-subjects behavioral manipulation, except for the experiment depicted in a figure (see Fig. 3*C–E*), which was a full within-subjects design. All animals were exposed to at least four contexts during the experiment in which they participated. Animals in the within-subjects behavioral designs were exposed to a total of six contexts. All contexts were assigned pseudorandomly to each experimental phase, except for contexts 5, 7, and 8, which were used exclusively to habituate animals to the objects presented as contextually novel during the practice phase. All animals that underwent the retrieval practice paradigm went through a shaping phase (see explanation below) and then started the experiment.

Arena 1 was 50 cm wide × 50 cm long × 39 cm high with black plywood walls and floor, divided into nine squares by white lines. Arena 2 was an acrylic box 60 cm wide × 40 cm long × 50 cm high. The floor was white as well as two of its walls, which had different visual cues, geometric forms, or strips made with self-adhesive paper tape of different colors. The front wall was transparent, and the back wall was hatched. Arena 3 was 50 cm in diameter × 50 cm high, round with brown acrylic walls and black plywood floor, divided into nine squares by white lines. Arena 4 was a box 50 cm wide × 50 cm long × 40 cm high that was constructed with white Plexiglas. The floor was made of white Plexiglas as well. Each wall had different visual cues, geometric forms, or strips made with self-adhesive paper tape of different colors. Arena 5 was round, 40 cm in diameter × 50 cm high, with brown acrylic walls and sky-blue floor. Arena 6 was a bow-tie-shaped maze made of opaque white Plexiglas. The maze was 94 cm long, 50 cm wide, and 50 cm high. Each end of the apparatus was triangular, the apexes of which were joined by a narrow corridor (14 cm wide). Arena 7 was a Y-shaped apparatus constructed from Plexiglas. All walls were 40 cm high, and each arm was 27 cm in length and 10 cm wide. Arena 8 was an equilateral triangular 40 cm side × 40 cm high made of white semirigid PVC with a white floor made of the same material.

### Objects

All experiments used numerous junk objects, each differing in shape, texture, size, and color. The height of the objects ranged from 8 to 24 cm, and they varied with respect to their visual and tactile qualities. All objects had duplicates so that identical objects could be used at the same time. All objects were affixed to the floor of the apparatus with an odorless reusable adhesive to prevent them for being displaced during each session. Specific objects were never repeated across different conditions for a given animal. All objects were cleaned with 50% alcohol wipes after each session.

### Memory test for retrieval-induced forgetting

#### Overview

Rats as well as many other species innately prefer novel objects to familiar ones and, in displaying this preference, reveal memory for the familiar object (Berlyne, 1950; Ennaceur and Delacour, 1988; Thompson et al., 1991; Winters et al., 2008; Blaser and Heyser, 2015; May et al., 2016). As in our previous study, we capitalized on this tethering of innate behavior and cognition to show that remembering a prior encounter with one object caused rats to forget other objects seen in the same setting (Bekinschtein et al., 2018). We modified the spontaneous object recognition procedure to include three phases equivalent to the ones present in human studies of retrieval-induced forgetting (Anderson et al., 1994; Ciranni and Shimamura, 1999; Maxcey and Woodman, 2014; Wimber et al., 2015): encoding, retrieval practice, and test. In addition to this theoretically critical RP condition, there were two control conditions in which the intervening retrieval practice phase was replaced either by returning the rat to its home cage [time control (TC)] or by giving the rat the same number of exploration trials on entirely new objects [the interference control (IC)]. For each experiment, different cohorts of animals were used. For all the experiments (with the exception seen in Fig. 3*C–E*), animals were randomly assigned to one of the three possible conditions after the shaping phase (see below). The order in which they were exposed to each treatment (drug/vehicle or practice length) was pseudorandomly assigned, and experiments were conducted over a span of 2 weeks. Once we finished evaluating the animal for one of the treatments, we waited at least 4 d to start testing the other treatment. For the experiment described in Figure 3, *D* and *E*, animals were exposed to each condition (RP, IC, and TC). The order in which they were exposed to each condition was pseudorandomly assigned, and experiments were conducted over a span of 3 weeks. Once we finished evaluating the animal in one condition (e.g., retrieval practice) we waited 4 d to start testing the following condition (e.g., interference control).

##### The general retrieval practice paradigm.

Our new retrieval practice paradigm generally involved the following three conditions: RP, IC, and TC (Bekinschtein et al., 2018). All the conditions followed the same basic sequence across 3 d, as follows: day 1, habituation to the contexts; day 2, habituation to “distractor” objects to be used during the retrieval practice phase of the experiment; day 3, the main memory task (during the main memory task, encoding and practice phases took place in a single session); and day 4, test phases.

##### Habituation.

We incorporated a shaping procedure that included four sessions of object exposure. During shaping, rats were first habituated to two different contexts (10 min each, not described in the Apparatus section), and 3 h later rats were exposed to two pairs of novel objects in two contexts. The animals were exposed twice to each context (four sessions) with a delay of 20 min. In each session that lasted 5 min, the rats encountered the same two pairs of different objects in distinct locations. The objects were novel during the first exposure, but familiar during the next three. Each rat saw the four objects twice in both contexts. For each context, the location of the objects was different between the first and the second exposure. The shaping phase was conducted only once during the first week of the experiment independently of the condition assigned for that particular week. We added this procedure to familiarize rats with the possibility that the very same objects could be presented in different locations within a context or across contexts (Bekinschtein et al., 2018). All experiments started 72 h after shaping.

On the first day of the experiment, animals were habituated to two arena contexts (e.g., contexts 1 and 2) and were allowed to explore each context for 10 min. On the second day, each animal was exposed to three pairs of identical novel objects (X, Y, and Z) in context 2 in three consecutive (30 min apart) sessions, for 5 min each. The following day, the task was conducted in context 1.

##### RP condition.

The sample phase consisted of two consecutive sessions separated by 25 min. In these sample sessions, the animal was allowed to freely explore for 5 min two identical copies of two novel objects [e.g., Object A (session 1) and Object B (session 2)]. The practice phase took place 60 min after the last sample session. This phase consisted of three 3 min sessions with an intersession interval of 15 min. In each session, the animal was exposed to a copy of one of the two encoded objects (e.g., Object A) presented during the sample phase, accompanied by one copy of objects X, Y, or Z, respectively, across the three trials (e.g., A and X; then A and Y; then A and Z across the three sessions). We pseudorandomly assigned which object was presented during the retrieval practice phase from the two objects that were sampled in the sampling phase (either A or B), so the practiced object could either be the first or the second one that was encoded in the sampling phase. Moreover, the location (right or left) in which the studied object appeared during retrieval practice was randomly assigned for each trial. The test phase was conducted 24 h after the last practice session. The animal was exposed for 3 min to a copy of a nonpracticed competitor object presented only during the sample phase (e.g., Object B) and one completely novel object (Object C). Thirty minutes later the animals were reintroduced to the context and exposed for 3 min to a copy of a practiced object (Object A) and one completely novel one (Object D). These two test sessions are defined in the Results section as a “competing object” and a “practiced object,” respectively. For both test sessions, the locations of the novel and familiar objects (right or left) were randomly assigned. The letters used in these descriptions and in our diagrams meant to indicate the nature of the item **(**i.e., the practiced object, competitor object, novel object, or distractor). Repetitions of the same letter across conditions do not indicate that the same object was used across conditions: in fact, different objects were used for the different conditions—RP, IC, or TC—of the task. Thus, Object A used in the RP condition is different from Object A used in the IC or TC conditions.

##### IC condition.

On the first day, the animals were habituated to two contexts (e.g., contexts 3 and 4) and allowed to explore them for 10 min each. On the second day, each animal was exposed to three novel objects (X, Y, and Z) in three consecutive sessions (30 min apart), and in context 4 for 5 min each. On the third day, the main memory task was conducted in context 3. On this final day, during the sample phase each rat was allowed to freely explore for 5 min two identical copies of two novel objects (Objects A and B) in two consecutive sessions separated by 25 min. The practice phase took place 60 min after the sample phase. During this phase, the animal was allowed to explore two copies of Objects X, Y, and Z in context 3 during three consecutive 3 min sessions with a delay of 15 min between each session. The test phase (24 h after the last practice session) consisted of a 3 min exposure to a copy of Object B and one completely novel object (Object C). The time the animals spent exploring the objects in each trial was manually recorded using hand chronometers. The order in which the sample objects were tested was pseudorandomly assigned, and the position in which the sample objects appeared on the final test was randomly determined.

##### TC condition.

On the first day, the animals were habituated to one context (e.g., arena context 5), and allowed to explore it for 10 min. On the second day, the animals were transferred to the behavioral testing room but were allowed to stay in their home cage for the duration of time that the animals assigned to the other two conditions were habituated to the novel objects. On the third day, the main memory task was conducted in context 5. The sample phase consisted of two consecutive sessions separated by 25 min. In these sessions, the animal was allowed to freely explore for 5 min two identical copies of two novel objects: Object A (session 1) and Object B (session 2). Unlike in the RP and IC conditions, however, there were no practice trials; instead, the rats spent the same interval of time in their home cages in between the sample phase and the test. The test phase took place 24 h later. During this phase, the animal was exposed to a copy of Object B and a completely novel object (Object C) for 3 min. The order in which the sample objects were tested was pseudorandomly assigned, and the position in which the sample objects appeared on the final test was randomly determined.

##### Quantification of behavior.

The behavioral responses of the animals for all experiments were analyzed given the following criteria. We defined exploration of an object as the rat directing its nose to the object at a distance of <2 cm and/or touching it with its nose. Turning around or sitting on the object was not considered exploratory behavior. Encoding, practice, and test phases were recorded using cameras (model HMX-F80, Samsung). The cameras were located on top of each arena, allowing the visualization of the complete space. Offline analysis was performed using Stopwatch software (Center for Behavioral Neuroscience, Emory University, Atlanta, GA) by a trained person. The test phase was analyzed by an experimenter who was blind to the conditions of the experiment.

Based on these criteria, we calculated a discrimination index (DI) for each trial of each session on each condition, as follows.

###### Practice trials

A discrimination index was calculated as the difference in time spent exploring the contextually novel and familiar objects divided by the total time spent exploring the objects (i.e., [(contextually novel – familiar)/total exploration time]).

###### Test trials

A discrimination index was calculated as the difference in time spent exploring the novel and familiar objects divided by the total time spent exploring the objects (i.e., [(novel – studied)/total exploration time]). In our experiments, we treat discrimination indices that exceed 0 in a given condition as evidence for memory of the previously presented object, as is common with the spontaneous object recognition procedure (for a detailed consideration of alternative factors that may contribute to this measure, see the study by Gulinello et al., 2019). The process of retrieval-induced forgetting is evidenced by lower discrimination index scores (i.e., worse memory) of the competitor object in the RP condition compared with the IC and TC conditions in which there is no retrieval practice.

##### Criteria of exclusion.

Animals that explored the objects for <10 s during any of the phases were excluded from the experiments. However, no rats had to be excluded from the study based on this criterion.

### Specific design features of individual experiments

#### Surgery and drug infusions

Rats were deeply anesthetized with ketamine (60 mg/kg) and xylazine (8 mg/kg) and put in a stereotaxic frame (Stoelting). The skull was exposed and adjusted to place bregma and λ on the same horizontal plane. After small holes were drilled, a set of 22 g guide cannulae were implanted bilaterally into the mPFC [anteroposterior (AP), +3.20 mm; left lateral (LL), ±0.75 mm; dorsoventral (DV), −3.50 mm] and/or the VTA (AP, −7.20 mm; LL, ±0.75 mm; DV, −5.30 mm; Paxinos and Watson, 1998). Cannulae were fixed to the skull with dental acrylic. A dummy cannula was inserted to each cannula to prevent clogging. At the end of surgery, animals were injected with a single dose of meloxicam (0.2 mg/kg) as an analgesic and gentamicin (0.6 mg/kg) as antibiotic.

Behavioral procedures commenced 5–7 d after surgery. On the experimental day, the dummy cannulae were removed before the injection and an injection cannula extending 1 mm below the guide cannula was inserted. The injection cannula was connected to a 10 µl Hamilton syringe. Cannulated rats received bilateral 0.5 µl infusions of the corresponding drug/vehicle. Muscimol (Mus; 0.1 mg/ml in saline; catalog #2763–96-4, Sigma-Aldrich) infusions into the VTA occurred 15 min before the retrieval practice phase. Injections were also made before exposure to the interpolated objects (equivalent to the “practice phase”) in the IC condition or before returning rats to their home cages for the TC condition.

SCH 23389 (SCH; 3 mg/ml in saline, 0.5 µl/side; catalog #0925/10, Tocris Bioscience) and SKF 38393 (SKF; 8.41 mg/ml in saline, 0.5 µl/side; catalog #0922/100, Tocris Bioscience) occurred 10 min before the retrieval practice phase (or at the corresponding points in TC conditions). We conducted the final test 24 h later. Doses were chosen based on previous studies (Gonzalez et al, 2014) and solubility data.

#### Cannulae placement

To check cannulae placement, 24 h after the end of the behavioral experiments, animals were infused with 1 µl of methylene blue through the dummy cannulae, and 15 min later were deeply anesthetized and killed. Histologic localization of the infusion sites was established using magnifying glasses. Five animals were excluded because of cannulae misplacement.

To control for VTA coordinates of infusions, three stereotaxically cannulated rats were infused with Green Beads (1:1000 dilution of concentrated 1-μm-diameter fluorescent beads; Bangs Laboratories). Seven days after the infusion, animals were deeply anesthetized with ketamine/xylazine and transcardially perfused with 10 ml of 0.04% heparin cold saline followed by 20 ml of 4% paraformaldehyde in 0.1 m PBS. Brains were removed and immersed overnight in the same fixative. Then brains were stored in a 0.1 m PBS 30% sucrose solution at 4°C until processed.

#### Immunohistochemistry assay

Thirty-five-micrometer-thick coronal brain sections were cut in a cryostat (Leica). Sections containing the VTA region were preserved in 0.1 m PBS. Dopamine neurons were confirmed by immunohistochemical detection of tyrosine hydroxylase (TH). Briefly, sections were blocked for 2 h at room temperature and then incubated with mouse anti-TH antibody (1:1000; catalog #MAB318, Sigma-Aldrich) overnight at 4°C, washed three times, and incubated with a conjugated Cy3 goat anti-mouse secondary antibody (1:500; catalog #115–165-146, Jackson ImmunoResearch) for 2 h at room temperature. Finally, the slides were incubated with DAPI and mounted.

### Experimental design and statistical analysis

**Figure 1.**
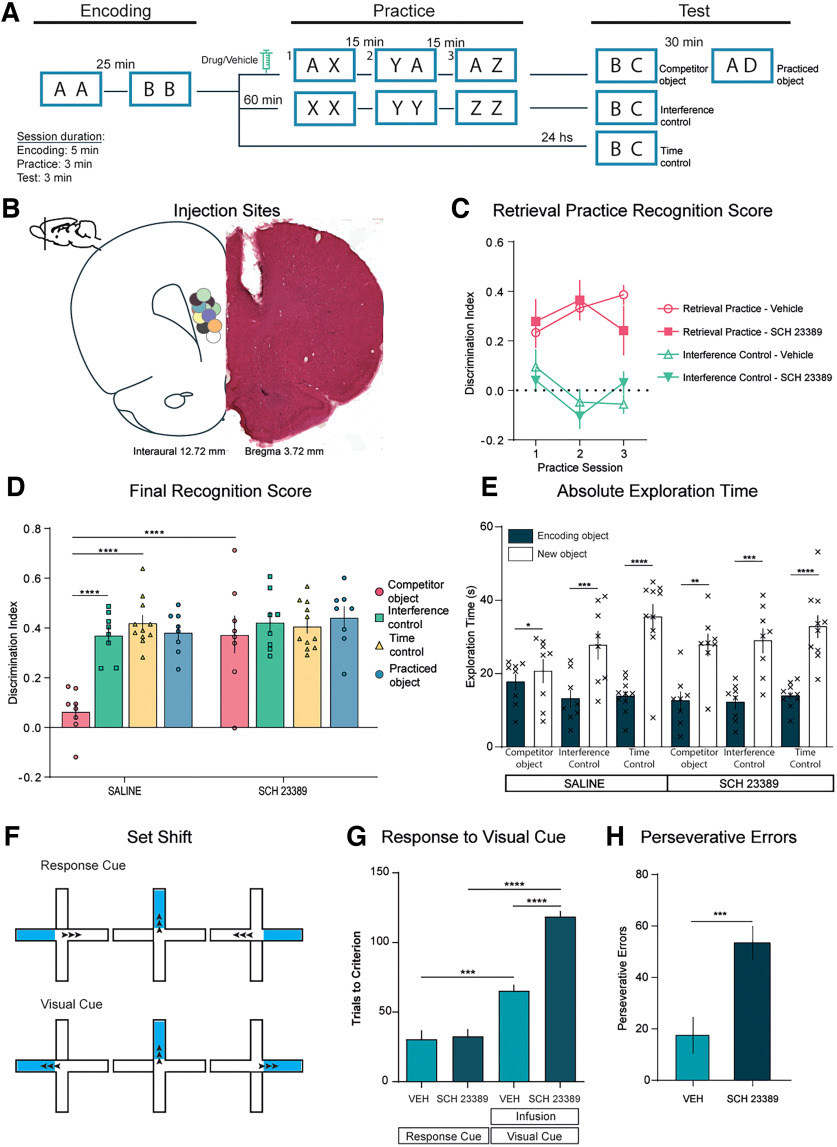
D_1_ receptors in medial prefrontal cortex mediate retrieval-induced forgetting. ***A***, Schematic representation of the behavioral protocol. After the acquisition, animals were divided into the three different conditions, RP, IC, and TC. The syringe indicates the infusion of the drug or its vehicle 10 min before the practice phase. Animals were assigned to one condition and subjected to a different pharmacological treatment each week. ***B***, Histology. Left, Diagram of the coronal section of the rat brain, showing the placement of the markings produced by methylene blue infusion for all the rats that received infusions of dopaminergic (or vehicle) drugs in the mPFC. The sections of the brain correspond to the atlas by Paxinos and Watson (1998). Right, Safranin staining showing an example of the cannula track left. ***C***, Discrimination indexes for the three sessions of the practice phase for the RP and IC groups in drug conditions and their vehicle (Table 1). ***D***, Discrimination indexes ± SEM for the testing phase. Animals performed the task twice, once with the drug and once with the vehicle in a pseudorandomized way and for the same condition (Table 2), two-way ANOVA, *n* = 8–11, Bonferroni's *post hoc* comparisons are shown indicated by asterisks. ***E***, Exploration times ± SEM for each individual object in the test phase (Table 2) compared by a paired *t* test, shown with asterisks. ***F***, Training schemes for the set-shifting task, with the Response Cue (left, egocentric) and the Visual Cue (right, visual). The arrows indicate the correct turn expected for each example trial. ***G***, Trials to criterion ± SEM are the number of trials conducted to complete a Criterion test correctly. Ordinary one-way ANOVA, *n* = 5; Tukey's *post hoc* comparisons are shown indicated by asterisks. ***H***, Perseverative errors ± SEM, each trial in which the animal responded according to the self-centered key. Perseverative errors were defined as entering the wrong arm in three or more trials per block. Unpaired *t* test comparisons are shown by asterisks. *p < 0.05, **p < 0.01, ***p < 0.001, and ****p < 0.0001.

**Figure 2.**
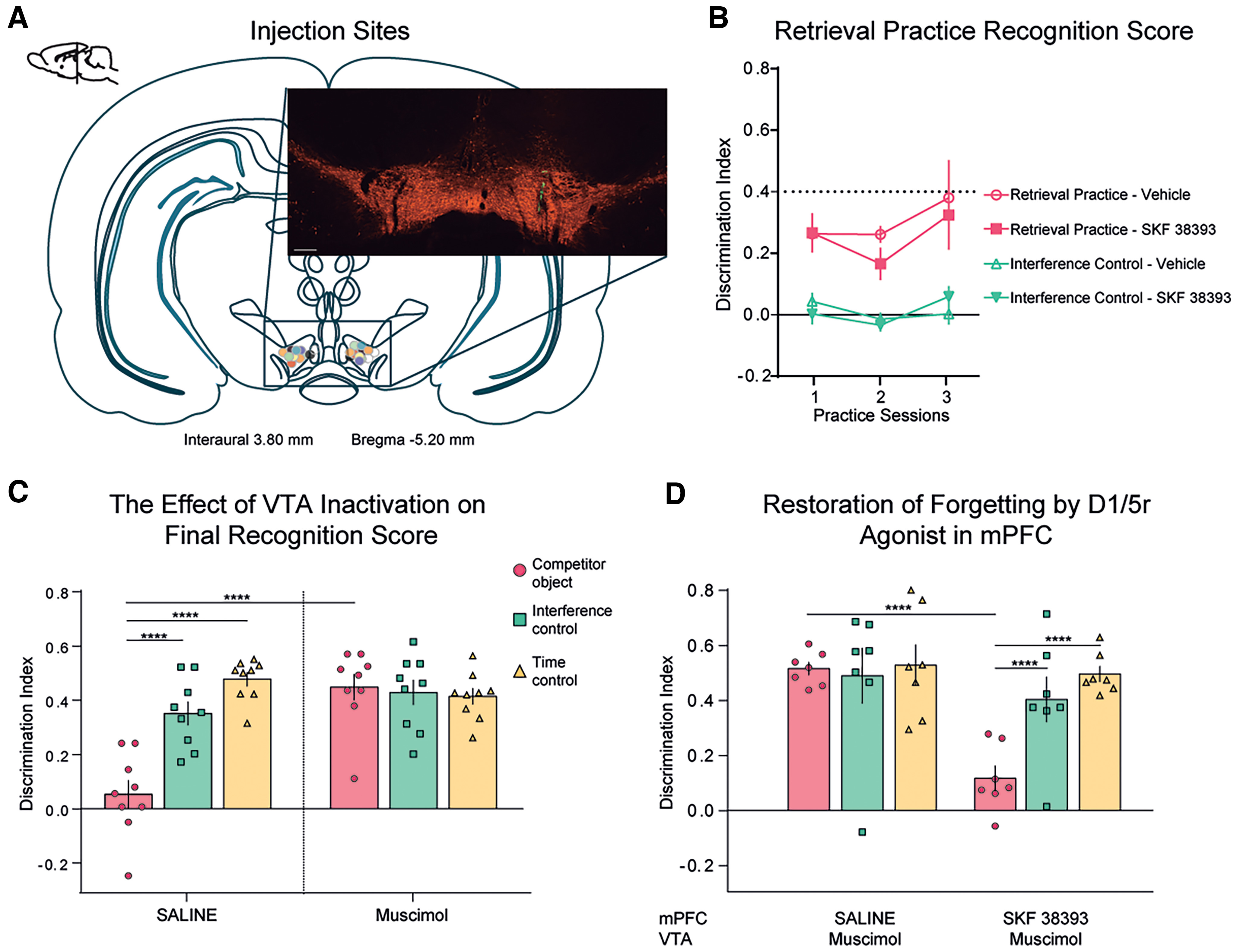
VTA projections to mPFC are necessary for retrieval-induced forgetting. ***A***, Diagram of the coronal section of rat brain, showing the site of infusion of fluorescent green beads for all rats injected with muscimol (or vehicle) in the VTA. The sections of the brain correspond to the atlas by Paxinos and Watson (1998). The orange immunofluorescence corresponds to TH detection; the green color corresponds to green beeds infused through the implanted cannulae. Scale bar, 200 μm. ***B***, Discrimination indexes ± SEM for the three sessions of the practice phase for the RP and IC groups under both conditions. ***C***, Discrimination indexes ± SEM for the test phase after Mus or Veh injection into the VTA. Two-way ANOVA with Bonferroni's corrected *post hoc* analysis. There was a significant drug × condition interaction. Muscimol impaired the forgetting of the competitor object. ***D***, Discrimination indexes ± SEM for the test phase of the “restoration of forgetting” experiment by infusion of SKF 38393 in mPFC. The animals performed the task twice, once with the drug and once with the vehicle in a pseudorandomized way for the condition to which they were pseudorandomly assigned after the training phase. All animals were infused with muscimol in the VTA. Two-way ANOVA followed by a Bonferroni's *post hoc* analysis indicated a significant drug × condition interaction. **p < 0.01, ***p < 0.001, and ****p < 0.0001.

**Figure 3.**
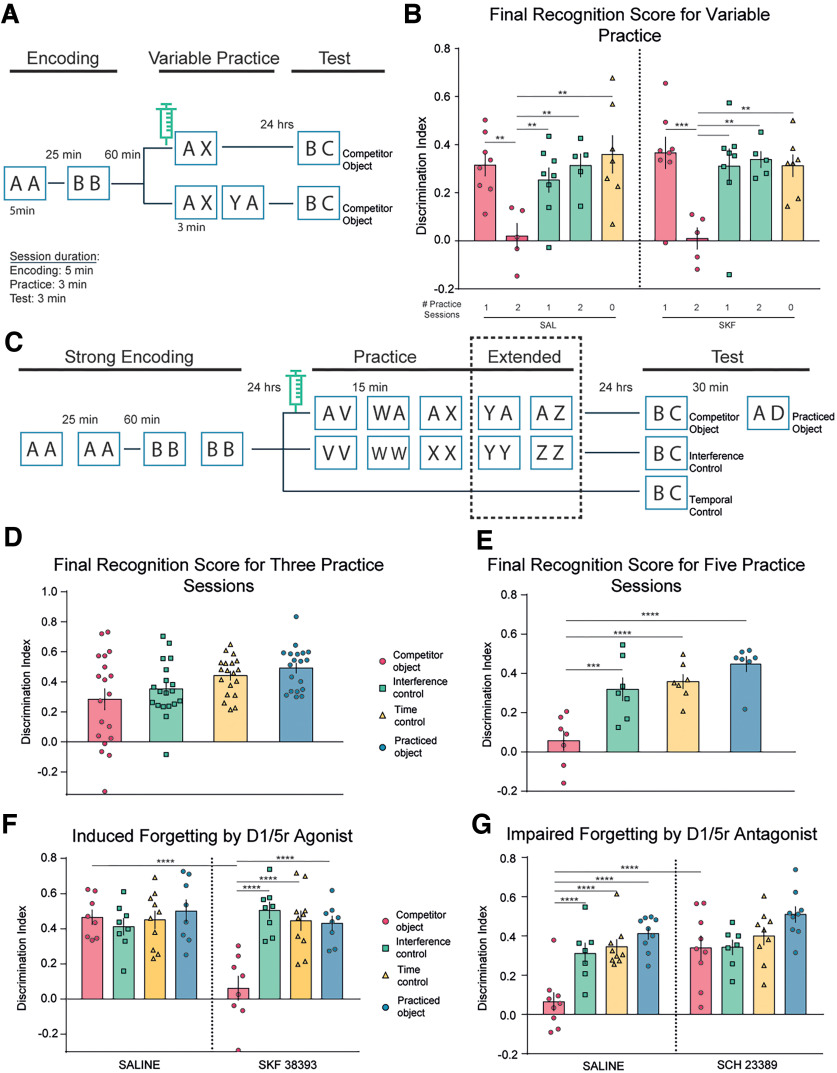
Bidirectional modulation of retrieval-induced forgetting. ***A***, Schematic representation of the behavioral protocol. After the acquisition, the animals were divided into three conditions: RP, IC, and TC. Both RP and IC were subdivided into two groups: (1) a group that performed a practice phase with only one retrieval practice session and (2) another group that did two retrieval practice sessions. Only the RP group is schematized; the IC group performed the equivalent to the practice phase with two copies of identical objects (XX or XX and then YY). The syringe indicates the infusion of SKF or its vehicle (saline) 10 min before the practice phase. ***B***, Discrimination rates for the test phase. The animals performed the task twice, once with the SKF and once with saline in a pseudorandomized way and for the same condition. Two-way ANOVA followed by a Bonferroni's *post hoc* test. ***C***, Schematic representation of the behavioral protocol. The protocol consisted of an acquisition phase with double training for each object (strong acquisition). ***D–G***, After the acquisition, the animals were divided in three conditions, RP, IC, and TC; the top panels (***D***, ***E***) correspond to two groups of animals that performed the protocol without infusion of any drug, and the bottom panels (***F***, ***G*)** correspond to the other two groups of animals that were cannulated and infused with the D_1_R agonist and antagonist. The syringe indicates the infusion of the drug or its vehicle 10 min before the practice phase (extended practice, one-way ANOVA for ***D*** and ***E***, and two-way ANOVA for ***F*** and ***G***). *p < 0.05; **p < 0.01; ***p < 0.001; and ****p < 0.0001.

Statistical analyses were performed using GraphPad version 6.01. In the experiments in which we used drug infusions, the “drug” variable was analyzed within a subject and the “condition” variable (i.e., RP, IC, and TC), between subjects. Each subject was tested in one condition, with vehicle and with drug, in a pseudorandomized way. This type of analysis corresponds to the experiments from Figures 1*C*, 2*C*, and 3*B*, *F*, and *G*. For the experiment in Figure 3*B*, each subject was assigned a single condition (one practice or two practice sessions were treated as distinct conditions). For the experiments that did not involve drug infusions, the “condition” variable was analyzed within subject; each subject experienced all three conditions in a pseudorandomized way, which this corresponds to experiments in Figure 3, *D* and *E*. For all retrieval-induced forgetting experiments, individual object exploration times during test phase were analyzed using a paired *t* test (see tables). Discrimination indexes calculated from the test phase object exploration times were analyzed using a two-way repeated-measures ANOVA followed by Bonferroni's *post hoc* comparisons in the experiments with drugs or vehicle and using a repeated-measures one-way ANOVA followed by Bonferroni's *post hoc* comparisons in the experiments without drug or vehicle infusion (in Results). Asterisks shown in graphs represent *p*-values for the *post hoc* analysis (**p < 0.01, ***p < 0.001, and ****p < 0.0001). In all cases, *p*-values were considered to be statistically significant at *p* < 0.05. Discrimination indexes calculated from the retrieval practice phase sessions object exploration times were analyzed using one-tailed unpaired *t* test (see tables; total exploration times are compared in Results). Absolute exploration times between vehicle-infused and drug-infused animals for each retrieval practice session (e.g., RP group: drug A + X mean vs vehicle A + X mean; IC group: drug X1 + X2 mean vs vehicle X1 + X2 mean) were compared using an unpaired Student's *t* test (see tables).

**Table 1.** Exploration times and discrimination indexes during the practice phase in the retrieval practice condition for experiment depicted in Figure 1*A*

	Saline			SCH 23389			*p*_total_	*n*
	A	X/Y/Z	DI	A	X/Y/Z	DI		
RP								
S1	9.45 ± 1.67	18.12 ± 3.65	0.23 ± 0.06	9.89 ± 1.55	15.12 ± 1.63	0.27 ± 0.08	0.77	9
S2	6.39 ± 2.44	13.02 ± 3.78	0.33 ± 0.04	7.16 ± 1.29	14.23 ± 2.34	0.36 ± 0.08	0.14	9
S3	4.18 ± 0.51	8.59 ± 1.61	0.38 ± 0.03	4.23 ± 0.56	9.39 ± 1.14	0.24 ± 0.10	0.42	9
	X/Y/Z	X/Y/Z	DI	X/Y/Z	X/Y/Z	DI	*p*_total_	*n*
IC								
S1	8.84 ± 1.78	9865 ± 1.94	0.09 ± 0.07	9.53 ± 1.75	10.48 ± 1.53	0.04 ± 0.04	0.30	10
S2	8.10 ± 0.89	6.94 ± 0.842	−0.04 ± 0.05	13.50 ± 1.87	13.69 ± 3.09	−0.10 ± 0.05	0.81	10
S3	5.01 ± 1.04	5.36 ± 1.02	−0.05 ± 0.03	10.39 ± 1.97	9.52 ± 1.89	0.025 ± 0.04	0.06	10

Retrieval practice phase. Total exploration times during the retrieval practice phase and DI for the RP and IC groups when animals were infused with saline (left) or SCH 23389 (right). Values are expressed in seconds (mean ± SEM). Student's t test, comparing DI between saline- and SCH-injected animals for each retrieval practice session (e.g., SCH 23389 A + X mean vs saline A + X mean for the RP group and SCH 23389 × 1 + X2 mean vs saline X1 + X2 mean, for IC group). Significance level is indicated as *p*_total_. SCH 23389 injection did not affect total exploration times during the practice phase compared with saline injection.

**Table 2.** Exploration times during the final test phase for experiment depicted in Figure 1*A*

	Saline				SCH 23389					
	Object B	Object C	*p*	Total	Object B	Object C	*p*	Total	*p*_total_	*n*
RP–	17.56 ± 2.25	20.84 ± 3.24	0.0562	38.4 ± 5.43	12.74 ± 2.44	27.96 ± 3.02	****	40.69 ± 4.39	0.72	8
IC	13.25 ± 2.38	27.95 ± 3.80	****	41.2 ± 6.03	12.25 ± 1.81	29.11 ± 3.33	****	41.36 ± 4.88	0.97	8
TC	13.93 ± 1.43	35.62 ± 3.48	****	49.55 ± 4.63	14.03 ± 1.04	32.95 ± 3091	****	46.98 ± 3.80	0.54	11
RP+*	13.51 ± 2.05	28.6 ± 3.42	****	42.11 ± 5.26	11.34 ± 2.40	28.58 ± 4.97	****	39.93 ± 6.97	0.77	8

Absolute exploration times during the final test phase. Total exploration times during the final test phase for the RP–, IC, TC, and RP+ conditions. Values are expressed in seconds (mean ± SEM). Paired Student's *t* test, comparing individual object exploration time between saline- and SCH-injected animals for the test phase; significance level is indicated as *p*. Paired Student's *t* test, comparing total exploration time between saline- and SCH-injected animals for the test phase (e.g., SCH 23389 B + C mean vs saline B + C). Significance level is indicated as *p*_total_.

*RP+ group was exposed to the practiced object A.

****p < 0.0001.

For the set-shifting experiment, the Acquisition criterion and Trials criterion were analyzed using a one-way ANOVA; Tukey's *post hoc* comparisons are indicated by asterisks. Comparisons between response and visual conditions were made considering the response group as two distinct groups (two groups of *n* = 5 each), segregating the animals that later performed the visual cue training with vehicle or with SCH (in Results) and comparing, respectively. Total Perseverative Errors, Perseverative Errors, Regressive Errors, and Never Reinforced Errors were compared for the visual cue training between the vehicle and SCH treatments using an unpaired *t* test (see tables). For data details, see the tables.

### Set-shifting task

#### Apparatus

The cross-maze was a four-arm maze made of 1-cm-thick black Plexiglas (Fig. 1*F*). The maze was placed on the floor. Each arm was 52 cm long and 9 cm wide; the height of the arm wall was 40 cm. Each arm contained a food well (diameter, 3 cm; height, 2.5 cm) that was 3.2 cm from the end wall.

#### Habituation procedure

The habituation procedure was similar to that described in the study by Ragozzino (2002). Rats were allowed 7–10 d to recover from surgery before the habituation procedure commenced. Rats were food restricted to 85% of their original *ad libitum* weight. During food restriction, rats were handled for 10 min/d. On the first day of habituation, three pieces of Fruit Loops cereal (Kelloggs) were placed in each arm, with two pieces in the food well. A rat was placed in the maze and allowed to freely navigate and consume cereal pieces for 15 min. If a rat consumed all 12 cereal pieces before 15 min, then the rat was placed in a holding cage, the maze was rebaited, and the rat was placed back in the maze; this process was repeated a total of three times (if a rat did not consume all 12 cereal pieces before 15 min, then the habituation day 1 was repeated the next day until the rat reached criterion). On the second habituation day, the procedure was similar except that after a rat consumed two cereal pieces per arm, the rat was picked up and placed in a different arm. This acclimated the rat to being handled in the maze after consuming cereal. On subsequent habituation sessions, the procedure was the same as that on day 2, except that there were only two half-pieces of cereal put in each food well. Each time a rat consumed all the cereal pieces after being placed in the maze was considered one trial. This procedure continued until a rat consumed cereal from all food wells for four trials or more in a 15 min session. On the last day of habituation, the turn bias for a rat was determined. The maze was arranged such that a white Plexiglas block (9 × 40 × 1 cm) was placed at the center entrance of one of the arms so that it prevented entry into that arm, giving the maze a T shape. A rat was started from the stem arm and allowed to turn left or right to obtain a half-piece of cereal. In one of the choice arms, a white-blue piece of posterboard (8 × 48 × 0.3 cm) was placed on the floor (Fig. 1*F*). After a rat made a turn and consumed a cereal piece, the rat was picked up, placed in the stem arm, and allowed to make a choice. If the rat chose the same arm as in the initial choice, it was returned to the stem arm until it chose the other arm and consumed the cereal piece. After choosing both arms, the rat was returned to the holding cage, the block and visual cue were moved to different arms, and a new trial was begun. Thus, a trial for the turn-bias procedure consisted of entering both choice arms and consuming both cereal pieces. This procedure continued for seven trials. The turn that a rat made first during the initial choice of a trial was recorded and counted toward its turn bias. Whatever direction (right or left) a rat turned, four or more times during these seven trials was considered its turn bias. During response discrimination testing, a rat was required to turn in the opposite direction of its turn bias. Behavioral testing was started the next day.

#### Response–visual cue testing procedure

The testing procedure was similar to that described in the study by Ragozzino (2002) except that all testing was carried across two consecutive sessions. For each discrimination, three start arms were used. In this experiment, each rat started on the response version. A rat started from the arms designated west (W), south (S), and east (E), leaving the north (N) arm unused as a starting arm. The visual cue was placed pseudorandomly in one of the choice arms such that for every consecutive set of 12 trials it occurred an equal number of times in each choice arm. During the acquisition session, a rat had to turn in the opposite direction of its turn bias to receive a half-piece of Froot Loops cereal. Figure 1*F* (top) illustrates an example of the correct navigation patterns for a rat that was required to always make a turn to the right. Between trials, a rat was placed back in the holding cage, which sat on a shelf next to the maze. The intertrial interval was <20 s. To minimize the use of intramaze cues from the apparatus, every six trials the maze was turned 90° clockwise relative to the experimenter. A rat reached criterion when it made 10 correct choices consecutively. There was no limit for the number of trials prearranged for a rat to reach this criterion. Once a rat made 10 correct choices consecutively, a probe trial was given. The probe trial consisted of starting the rat from the fourth arm (N) that was not used during testing. If a rat correctly turned the same direction as on testing, then the response procedure was completed. If a rat made an incorrect turn, then response testing was continued until a rat made an additional five correct choices consecutively, at which time another probe trial was administered. This procedure was continued until a rat made a correct choice on the probe trial. The following measures were taken for each rat: (1) acquisition criterion, defined as the total number of test trials to complete 10 consecutive correct choices in a session; (2) trials to criterion, defined as the total number of test trials completed before a correct choice on the probe trial was made; and (3) probe trials, defined as the total number of probe trials to get one correct. The day after reaching criterion on the response version, rats were switched to the visual cue version. Each rat was injected with SCH or Veh into the mPFC 15 min before the beginning of the visual cue learning session. In the visual cue version, a similar procedure was used as in the response version. However, in this test the rat always had to enter the arm with the visual cue. The visual cue was pseudorandomly varied in the left and right arms such that it occurred in each arm an equal number of times for every consecutive set of 12 trials. Figure 1*F* (bottom) shows an example of a rat that learned to always enter the visual cue arm. A rat reached criterion when it made 12 correct choices consecutively. There was no limit on the number of trials a rat was allotted to reach this criterion. Once a rat made 12 correct choices consecutively, a probe trial was given. If a rat correctly turned following the visual cue, then the response procedure was completed. If a rat made an incorrect turn (error), then visual testing was continued until a rat made an additional six correct choices consecutively, at which time another probe trial was administered..

Additional parameters were analyzed on the switch to determine whether treatments altered perseveration. Perseveration involved continuing to make the same egocentric response as required on the response version, when the trial required turning the opposite direction to enter the visual cue arm. For every consecutive 12 trials in a session, half the trials consisted of these trials. These trials were separated into consecutive blocks of four trials each (Ragozzino, 2002). Perseveration was defined as entering the incorrect arm in three or more trials per block. This is a similar criterion as used in previous experiments measuring perseveration (Ragozzino et al., 1999; Floresco et al., 2006). Once a rat made less than three errors in a block the first time, all subsequent errors were no longer counted as perseverative errors.

## Results

To test whether control processes regulated by dopamine in the mPFC participate in adaptive forgetting, we studied how exploratory behavior in a rodent object recognition task was affected by manipulation of the dopaminergic system.

The D_1_R is one of the main dopamine receptors in the mPFC (Sawaguchi and Goldman-Rakic, 1991; Arnsten, 1998). Thus, in experiment 1 we studied the role of mPFC D_1_Rs in retrieval-induced forgetting. Each rat, assigned to the RP, IC, or TC condition, was tested twice: once with saline and once with the D_1_R antagonist SCH. We injected SCH into the mPFC bilaterally (Fig. 1*B*) 10 min before the first retrieval practice session, and at the same time point in the IC and TC conditions. Thus, in drug studies, treatment (drug or saline) was done within subject, but the condition (RP, IC, or TC) was compared between subjects.

Infusing animals with saline or SCH did not alter their total exploration times during the retrieval practice phase (total exploration times: RP Veh, 51.12 s ±5.74; RP SCH, 59.21 s ±6.02; *n* = 9; paired *t* test: *p* = 0.24, *t* = 1.27, df = 8). For the RP group, both the saline and SCH treatments rats preferred the novel objects during practice trials, indicating that retrieval of the practiced object was not affected by SCH infusion (Fig. 1*C*, Table 1). Although SCH injection could have affected retrieval practice performance, we observed no evidence of this in any of the conditions.

On the final test, we scored the time rats spent exploring the old object versus the novel object (Fig. 1*E*). Our dependent variable was a discrimination index that reflects the bias in the time they spent exploring the novel item instead of the old one (Fig. 1*D*). If the discrimination index at test was significantly lower for the RP condition compared with the IC and TC conditions, we considered there was significant retrieval-induced forgetting. We found that saline-infused rats explored the competitor Object B as if it was new, as shown by the lower discrimination index in the RP condition compared with the IC and TC groups (Fig. 1*D*, Table 2). Critically, however, rats infused with SCH showed high discrimination indexes (two-way ANOVA; Interaction: *p* = 0.0013, *F*_(3,31)_ = 6.65; Drug: *p* = 0.0008, *F*_(1,31)_ = 13.77; Condition: *p* < 0.0001, *F*_(3,31)_ = 10.05; Subjects: *p* = 0.356, *F*_(31,31)_ = 1.14). Bonferroni's corrected comparisons confirmed that the discrimination indexes of rats for competitors were lower when infused with saline than with SCH, which is consistent with the possibility that SCH had prevented competitors from being forgotten. Indeed, infusing SCH abolished evidence for retrieval-induced forgetting completely (Fig. 1*D*). The discrimination index in the RP group was indistinguishable from that of the IC or TC groups. It is worth mentioning that the pharmacological manipulations were made right before the retrieval practice phase, after encoding had concluded. Thus, all groups encoded the objects in the absence of any drug. Since memory was evaluated 24 h after the retrieval practice phase, it is very unlikely that the drug itself affected retrieval during the test phase. So, the animals from the three groups (RP, TC, and IC) should have attempted to retrieve Object B in the same motivational, attentional, and perceptual state. Any changes in memory at test had to be the product of what happened during the practice phase. This phase is very similar in the RP and IC conditions. In both cases, the animals are exposed to contextually novel objects. However, retrieval-induced forgetting is only observed in the RP condition. In addition, we did not observe any differences between saline-infused and drug-infused animals in exploration during the retrieval practice phase. This indicates that perception, motivation, attention, or reactivity to novelty were not altered by the drugs.

To verify that the dose of SCH we used for our experiments was sufficient to impair cognitive control in a nonmemory task, a different group of rats infused with SCH or Veh was evaluated in a set-shifting task that requires the organism to exert inhibitory control over the tendency to engage in a previously relevant behavioral strategy (Ragozzino et al., 1999; Birrell and Brown, 2000; Stefani et al., 2003). Blockade of D_1_R in mPFC has been shown to impair performance in the set-shifting task (Ragozzino, 2002; Floresco et al., 2006).

SCH-injected rats produced significantly more errors than Veh-injected animals in the probe trials and required significantly more trials to reach criterion (Fig. 1*G*, Table 3, Acquisition Criterion; one-way ANOVA; treatment: *p* < 0.0001, *F*_(3,16)_ = 57.09; Response vs Visual Veh, *p* < 0.0001; Response vs Visual SCH, *p* < 0.0001; Visual Veh vs SCH, *p* < 0.0001). In addition, animals infused with SCH increased the number of trials to achieve the criterion relative to vehicle-infused animals (Fig. 1*G*, Table 3, trials to criterion; one-way ANOVA; treatment: *p* < 0.0001, *F*_(3,16)_ = 95.25; Response vs Visual Veh, *p* < 0.0001; Response vs Visual SCH, *p* < 0.0001; Visual Veh vs SCH, *p* < 0.0001) and made a greater number of perseverative errors (Fig. 1*H*, Table 3; unpaired *t* test; perseverative errors: *p* = 0.0173, *t* = 2.990, df = 8; total perseverative errors: *p* < 0.0001, *t* = 9.856, df = 8). Thus, the blockade of D_1_R receptors in the mPFC impaired shifting from an egocentric strategy to a visual strategy. This treatment equally affected cognitive control and retrieval-induced forgetting.

**Table 3.** Set-shift parameters for the experiment depicted in Figure 1*F*

	Response cue			Visual cue			Response vs visual
	Vehicle	SCH 23389*^a^*	Statistics	Vehicle	SCH 23389	Statistics	Statistics
Acquisition criterion	30.40 ± 6.15	32.60 ± 4.82	*p* = 0.78 *t* = 0.28, df = 8*^c^*	55.67 ± 4.61	109.7 ± 3.59	*p* < 0.0001 *t* = 9.23, df = 8*^c^*	*p* < 0.0001 *F*_(3,16)_ = 57.09*^b^*
Trials to criterion	25.60 ± 3.73	25.20 ± 6.06	*p* = 0.95 *t* = 0.05, df = 8*^c^*	65.33 ± 4.07	118.5 ± 3.80	*p* < 0.0001 *t* = 9.54, df = 8*^c^*	*p* < 0.0001 *F*_(3,16)_ = 95.25*^b^*
Total perseverative errors				17.00 ± 2.77	53.80 ± 2.49	*p* < 0.0001 *t* = 9.85, df = 8*^c^*	
Perseverative errors				5.60 ± 0.97	35.20 ± 6.88	*p* = 0.017 *t* = 2.99, df = 8*^c^*	
Regressive errors				12.60 ± 2.54	18.40 ± 5.97	*p* = 0.3976 *t* = 0.89, df = 8*^c^*	
Never reinforced errors				8.00 ± 2.21	1.40 ± 0.51	*p* = 0.019 *t* = 2.91, df = 8*^c^*	
*N*	5	5		5	5		

Set-shifting parameters. Acquisition criterion, defined as the total number of test trials to complete 10 consecutive correct choices in a session. Trials to criterion, defined as the total number of test trials completed before a correct choice on the probe trial was made. Probe trials, defined as the total number of probe trials to get one correct. Perseveration involved continuing to make the same egocentric response, as required on the response version, when the trial required turning the opposite direction to enter the visual cue arm. Perseveration was defined as entering the incorrect arm in three or more trials per block. After a rat stopped perseverating, the number of errors was counted when a rat reverted back to previously correct response (regressive errors) on those same types of trials that required the opposite turn as on the response version. Never reinforced errors were counted whenever a rat made an error by turning into the opposite response cue (with visual cue) arm.

*^a^*This group received vehicle infusion before the response cue training and SCH 23389 before visual cue training.

*^b^*One-way ANOVA.

*^c^*Unpaired *t* test.

The main prefrontal dopamine source is the VTA, which projects directly to the mPFC (Berger et al., 1991). We designed experiment 2 to establish whether dopamine release from VTA terminals into mPFC was required for retrieval-induced forgetting. We injected bilaterally Mus or Veh directly into VTA 15 min before the first retrieval practice session (Fig. 2*A*). Unlike permanent lesions, this treatment causes a transient silencing of the structure (Mao and Robinson, 1998), allowing the final object recognition test to occur in the absence of the drug.

Mus infusion in VTA did not affect total object exploration during the retrieval practice phase (total exploration times: RP Veh: 92.99 ± 9.35 s, *n* = 10; RP SCH: 85.97 ± 8.63 s, *n* = 12; unpaired *t* test: *p* = 0.58, *t* = 0.55, df = 20; Table 4). Critically, during the test phase, in the Veh-injected animals the discrimination index was significantly lower for the RP condition compared with the IC and TC groups, whereas we did not observe any difference between the RP and the control groups in Mus-infused animals (Fig. 2*C*; two-way ANOVA: Interaction: *p* < 0.0001, *F*_(2,48)_ = 16.29; Drug: *p* = 0.0002, *F*_(1,48)_ = 16.49; Condition: *p* < 0.0001, *F*_(2,48)_ = 11.95; Bonferroni's *post hoc* multiple comparisons) and exploration times (Table 5). Given that Mus had no effect in the IC or TC conditions (Fig. 2*C*, Table 5), this suggests that silencing the VTA did not modify recognition memory, but rather that VTA activity during the retrieval practice phase specifically affected object recognition at testing in the RP condition. These findings are consistent with our hypothesis that dopamine release into mPFC during selective retrieval practice was important for successful control processes that inhibited competing memories and produced retrieval-induced forgetting.

**Table 4.** Exploration times and discrimination indexes during the practice phase in the retrieval practice condition for experiment depicted in Figure 2, muscimol into the VTA

	Saline			Muscimol			*p*_total_	*n*
	A	X/Y/Z	DI	A	X/Y/Z	DI		
RP								
S1	11.31 ± 1.42	20.92 ± 4.21	0.26 ± 0.04	10.76 ± 1.56	22.04 ± 4.75	0.2657 ± 0.06	0.80	9
S2	10.11 ± 0.82	17.08 ± 1.03	0.26 ± 0.02	11.97 ± 1.86	15.54 ± 2.10	0.1652 ± 0.05	0.538	9
S3	8.91 ± 0.82	24.95 ± 3.23	0.38 ± 0.12	7.69 ± 1.44	17.86 ± 3.33	0.32 ± 0.11	0.32	9
	X/Y/Z	X/Y/Z	DI	X/Y/Z	X/Y/Z	DI	*p*_total_	*n*
IC								
S1	9.82 ± 1.23	10.85 ± 1.36	0.04 ± 0.028	12.84 ± 2.02	12.60 ± 2.30	0.002 ± 0.03	0.35	10
S2	12.46 ± 0.74	12.79 ± 0.81	−0.014 ± 0.02	15.75 ± 2.98	16.81 ± 3.13	−0.0342 ± 0.02	0.26	10
S3	12.89 ± 2.21	13.15 ± 2.03	0.002 ± 0.03	12.98 ± 1.50	14.85 ± 2005	0.05 ± 0.0344	0.74	10

Retrieval practice phase. Total exploration times during the retrieval practice phase and DI for the RP and IC groups when animals were infused with saline (left) or muscimol (right). Values are expressed in seconds (mean ± SEM). Unpaired Student's *t* test, comparing total exploration time between saline- and SCH-injected animals for each retrieval practice session (e.g., muscimol A + X mean versus saline A + X mean for RP group and Muscimol X_1_+X_2_ mean versus saline X_1_+X_2_ mean, for IC group). Significance level is indicated as *p*_total_. Muscimol injection did not affect total exploration times during the practice phase compared with saline injection.

**Table 5.** Exploration times during the final test phase for experiment depicted in Figure 2, muscimol into the VTA

	Saline				Muscimol				*p*_total_	*n*
	Object B	Object C	*P*	Total	Object B	Object C	*p*	Total		
RP–	23.85 ± 1.92	26.64 ± 3.23	0.041	49.49 ± 3.55	15.03 ± 1.82	39.73 ± 2.95	**	54.76 ± 3.84	0.32	9
IC	14.74 ± 1.91	27.62 ± 4.58	***	42.36 ± 5.87	15.58 ± 2.69	31.95 ± 3.27	***	47.53 ± 4.43	0.49	10
TC	13.35 ± 2.32	34.26 ± 5.90	****	47.61 ± 7.97	13.83 ± 1.34	29.9 ± 2.85	***	43.72 ± 3.60	0.66	8

Absolute exploration time during the final test phase. Total exploration times during the final test phase for the RP–, IC, and TC conditions. Values are expressed in seconds (mean ± SEM). Unpaired Student's *t* test, comparing individual object exploration time between saline- and Muscimol-injected animals for the test phase; significance level is indicated as *p*. Unpaired Student's *t* test, comparing total exploration time between saline- and muscimol-injected animals for the test phase (e.g., muscimol B + C mean vs saline B + C mean). Significance level is indicated as *p*_total_.

*p < 0,05;

**p < 0,01;

***p < 0,001;

****p < 0,0001.

In experiment 3, we sought to elucidate whether VTA projections to the mPFC were important to modulate activity in this structure and cause retrieval-induced forgetting; we thus combined Mus injections into the VTA with injection of the D_1_R agonist SKF into the mPFC in a new set of animals. Mus was injected bilaterally into the VTA in all animals 15 min before retrieval practice (or the equivalent phase in the IC and TC conditions). Injection of SKF or Veh into the mPFC was performed 10 min before retrieval practice (or the equivalent phase in the IC and TC conditions). SKF infusion did not produce any changes in exploration or recognition of the familiar object during the practice phase (total exploration times: RP Veh, 52.17 ± 8.50 s; RP SKF, 48.89 ± 4.14 s; unpaired *t* test: *p* = 0.73, *t* = 0.35, df = 12; Table 6). Critically, SKF administration into mPFC caused significant reductions in the discrimination index in the RP group on the final test, compared with Veh-infused animals. Thus, SKF completely reversed the effect of silencing VTA with Mus (Fig. 2*D*; two-way ANOVA; Interaction: *p* = 0.01, *F*_(2,36)_ = 4.59; Drug: *p* = 0.002, *F*_(1,36)_ = 10.84; Condition: *p* = 0.01, *F*_(2,36)_ = 4.72; Bonferroni's *post hoc* multiple-comparisons test) and exploration times at test (Table 7). No differences in discrimination indexes were found between Veh-infused and SKF-infused animals in the IC and TC groups (Fig. 2*D*, Table 7). To control that any fluid injection into the VTA could modify the mPFC response to SKF injection, we compared an RP group and an IC group when injecting Veh into the VTA and Veh or SKF into the mPFC. Again, the within-subject variable was the drug (Veh or SKF) and the between-subject variable was the condition (RP or IC). Both RP groups had lower discrimination indexes than the IC groups during the test phase, independent of the injected drug into the mPFC (RP Veh: 0.04 ± 0.01, *N* = 5; RP SKF: 0.07 ± 0.02, *N* = 5; IC Veh: 0.35 ± 0.05, *N* = 4; IC SKF: 0.52 ± 0.08, *N* = 4). There was a significant effect of the condition (*p* < 0.0001, *F*_(1,7)_ = 136.9, repeated-measures two-way ANOVA), but no interaction (*F*_(1,7)_ = 0.003, *p* = 0.95). Thus, in the absence of activity within the VTA, the activation of mPFC D_1_Rs was sufficient to lower the discrimination index selectively in the RP condition, suggesting that the activation of mPFC D_1_Rs via dopamine release from VTA is one of the main mechanisms required for retrieval-induced forgetting in rats.

**Table 6.** Exploration times and discrimination indexes during the practice phase in the retrieval practice condition for experiment depicted in Figure 2, muscimol into the VTA and SKF 38393 into the mPFC

	Muscimol VTA–saline mPFC			Muscimol VTA–SKF 38393 mPFC			*p*_total_	*n*
	A	X/Y/Z	DI	A	X/Y/Z	DI		
RP								
S1	7.57 ± 1.2	13.22 ± 2.51	0.22 ± 0.13	7.89 ± 0.64	14.18 ± 1.72	0.26 ± 0.08	0.74	7
S2	5.29 ± 1.56	10.28 ± 2.12	0.35 ± 0.10	4.90 ± 0.80	8.81 ± 1.65	0.23 ± 0.08	0.50	7
S3	4.79 ± 1.33	6.72 ± 1.42	0.21 ± 0.16	5.26 ± 2.34	7.84 ± 1.75	0.32 ± 0.20	0.68	7
	X/Y/Z	X/Y/Z	DI	X/Y/Z	X/Y/Z	DI	*p*_total_	*n*
IC								
S1	10.69 ± 1.36	11.12 ± 1.51	0.02 ± 0.04	9.46 ± 2.34	9.63 ± 2.14	0.06 ± 0.09	0.42	7
S2	6.67 ± 1.04	7.15 ± 1.00	0.03 ± 0.05	6.29 ± 0.76	7.53 ± 1.54	0.048 ± 0.10	0.88	7
S3	5.17 ± 0.73	4.98 ± 0.68	−0.01 ± 0.04	3.57 ± 0.48	4.50 ± 0.64	0.11 ± 0.03	0.58	7
	Saline VTA–saline mPFC			Saline VTA–SKF 38393 mPFC				
	A	X/Y/Z	DI	A	X/Y/Z	DI	*p*_total_	*n*
RP								
S1	7.57 ± 1.20	13.22 ± 2.51	0.22 ± 0.13	7.89 ± 0.64	14.18 ± 1.72	0.26 ± 0.08	0.74	5
S2	5.29 ± 1.56	10.28 ± 2.12	0.35 ± 0.10	4.90 ± 0.80	8.81 ± 1.65	0.23 ± 0.08	0.50	5
S3	4.79 ± 1.33	6.72 ± 1.42	0.21 ± 0.16	5.26 ± 2.34	7.84 ± 1.75	0.32 ± 0.20	0.68	5
	X/Y/Z	X/Y/Z	DI	X/Y/Z	X/Y/Z	DI	*p*_total_	*n*
IC								
S1	10.69 ± 1.36	11.12 ± 1.51	0.02 ± 0.04	9.46 ± 2.34	9.63 ± 2.14	0.06 ± 0.09	0.42	5
S2	6.67 ± 1.04	7.15 ± 1.00	0.03 ± 0.05	6.29 ± 0.76	7.53 ± 1.54	0.04 ± 0.10	0.88	5
S3	5.17 ± 0.73	4.98 ± 0.68	−0.01 ± 0.04	3.57 ± 0.48	4.50 ± 0.64	0.11 ± 0.033	0.58	5

Retrieval practice phase. Total exploration times during the retrieval practice phase and DI for the RP and IC groups when animals were infused with muscimol in the VTA and saline (left) or SKF 38393 (right) in the mPFC. Values are expressed in seconds (mean ± SEM). Unpaired Student's *t* test, comparing total exploration time between saline- and SCH-injected animals for each retrieval practice session (e.g., muscimol A + X mean vs saline A + X mean for RP group and muscimol X_1_ + X_2_ mean vs saline X_1_ + X_2_ mean, for IC group). Significance level is indicated as *p*_total_. Muscimol injection did not affect total exploration times during the practice phase compared with saline injection.

**Table 7.** Exploration times during the final test phase for experiment depicted in Figure 2, muscimol into the VTA and SKF 38393 into the mPFC

	Muscimol VTA–saline mPFC				Muscimol VTA–SKF 38393 mPFC				*p*_total_	*n*
	Object B	Object C	*p*	Total	Object B	Object C	*p*	Total		
RP–	7.36 ± 1.87	22.26 ± 4.71	***	29.62 ± 6.52	14.82 ± 1.38	18.00 ± 2.89	0.04	33.82 ± 4.13	0.17	7
IC	6.26 ± 0.94	22.15 ± 5.05	**	28.41 ± 5.78	8.24 ± 1.23	20.06 ± 3.80	***	28.30 ± 4.52	0.96	7
TC	8.52 ± 1.47	23.57 ± 2.99	***	32.09 ± 4.13	6.83 ± 1.72	23.88 ± 5.86	***	30.71 ± 4.41	0.55	7
	Saline VTA–saline mPFC				Saline VTA–SKF 38393 mPFC					
	Object B	Object C	*p*	Total	Object B	Object C	*p*	Total	*p*_total_	*n*
RP–	17.80 ± 1.48	19.60 ± 2.2	0.518	37.40 ± 3.68	15.12 ± 1.17	17.48 ± 1.56	0.262	32.60 ± 2.61	0.31	5
IC	10.25 ± 1.25	21.83 ± 3.45	*	32.08 ± 4.33	7175 ± 2.74	19.68 ± 3.75	*	26.85 ± 6.36	0.52	5

Absolute exploration time during the final test phase. Total exploration times during the final test phase for the RP–, IC, and TC conditions. Values are expressed in seconds (mean ± SEM). Unpaired Student's *t* test, comparing individual object exploration time between saline- and SKF-injected animals for the test phase; significance level is indicated as *p*. Unpaired Student's *t* test, comparing total exploration time between saline- and SKF-injected animals for the test phase (e.g., SKF 38393 B + C mean vs saline B + C mean). Significance level is indicated as *p*_total_.

*p < 0.05;

**p < 0.01;

***p < 0.001;

****p < 0.0001.

In humans, higher prefrontal dopamine availability has been associated with greater retrieval-induced forgetting (Wimber et al., 2011). To evaluate whether the activation of D_1_Rs improved retrieval-induced forgetting, we injected the D_1_R agonist SKF into mPFC in a new group of animals before a modified retrieval practice phase consisting of only one practice trial (Fig. 3*A*). We reasoned that whereas only one practice trial would likely be insufficient to produce retrieval-induced forgetting on its own, it might do so given the activation of D_1_Rs in mPFC, which could magnify the impact of inhibitory processes.

A single retrieval practice did not yield significant memory impairment during the later test phase either in the Veh-infused or SKF-infused animals (Fig. 3*A*, Tables 8, 9, 10; two-way ANOVA; Interaction: *p* < 0.90, *F*_(4,28)_ = 0.25; Drug: *p* = 0.71, *F*_(1,28)_ = 0.13; Condition: *p* < 0.0001, *F*_(4,28)_ = 10.06; Bonferroni's *post hoc* multiple-comparisons test). The impact of inhibition arising from one practice session may have not been strong enough to produce retrieval-induced forgetting. In prior work, we had already observed that exposure to two retrieval practice sessions during the practice phase produced retrieval-induced forgetting that was measurable in a test session 30 min after the practice phase comparing the RP and IC conditions (Bekinschtein et al., 2018). However, in the present study, the final test took place 24 h after the practice phase. Thus, we tested our hypothesis again, but with a protocol in which the animals were exposed to two practice sessions, as in our prior work (Bekinschtein et al., 2018), and injected with Veh or SKF (Fig. 3*A*). In this case, we found no differences between Veh-infused or SKF-infused animals in discrimination indexes on the final test, consistent with both groups showing similar and significant levels of retrieval-induced forgetting (Fig. 3*B*, Table 11). Decreasing the number of trials proved not to be a sensitive strategy to evaluate positive modulation of retrieval-induced forgetting.

**Table 8.** Exploration times and discrimination indexes during the practice phase in the retrieval practice condition for experiment depicted in Figure 3*A*, single practice session

	Saline			SKF 38393			*p*_total_	*n*
	A	X/Y/Z	DI	A	X/Y/Z	DI		
RP1								
S1	11.42 ± 1.66	17.53 ± 2.20	0.22 ± 0.03	16.25 ± 1.85	23.15 ± 3.38	0.1656 ± 0.05	0.18	8
	X/Y/Z	X/Y/Z	DI	X/Y/Z	X/Y/Z	DI	*p*_total_	*n*
IC1								
S1	18.03 ± 1.24	17.05 ± 1.74	−0.03 ± 0.02	18.44 ± 2.37	19.28 ± 2.58	0.02 ± 0.02	0.22	8

Absolute exploration times during the retrieval practice phase. Total exploration times during the retrieval practice phase and DI for the RP-1 and IC1 groups when animals were infused with SKF 38393 or saline in the mPFC. Values are expressed in seconds (mean ± SEM). Paired Student's *t* test, comparing total exploration time between saline- and SKF-injected animals for each retrieval practice session (e.g., saline A + X mean vs saline A + X mean for RP group and muscimol X_1_ + X_2_ mean versus saline X_1_ + X_2_ mean, for IC group). Significance level is indicated as *p*_total_. SKF 38393 injection did not affect total exploration times during the practice phase compared with saline injection.

**Table 9.** Exploration times and discrimination indexes during the final test phase for experiment depicted in Figure 3*A*, single practice session

	Saline				SKF 38393				*p*_total_	*n*
	Object B	Object C	*p*	Total	Object B	Object C	*p*	Total		
RP-1	15.38 ± 2.40	31.05 ± 3.65	****	46.43 ± 4.82	16.49 ± 2.65	31.86 ± 2.69	****	47.34 ± 4.65	0.76	8
IC1	16.03 ± 2.11	26.36 ± 1.45	***	42.39 ± 2.89	16.39 ± 3.15	28.01 ± 2.61	***	44.40 ± 4.95	0.73	8
TC	13.94 ± 2.79	24.19 ± 2.33	***	38.13 ± 4.91	13.02 ± 2.65	25.57 ± 2.33	***	38.59 ± 4.36	0.94	7
RP + 1	12.26 ± 2.14	36.52 ± 2.46	***	48.78 ± 3.29	10.78 ± 1.27	32.33 ± 5.13	***	43.11 ± 5.93	0.4265	8

Absolute exploration time during the final test phase. Total exploration times during the final test phase for the RP-1, IC1, and TC conditions. Values are expressed in seconds (mean ± SEM). Paired Student's *t* test, comparing individual object exploration time between saline- and SKF-injected animals for the test phase; significance level is indicated as *p*. Paired Student's *t* test, comparing total exploration time between saline- and SKF-injected animals for the test phase (e.g., SKF 38393 B + C mean vs saline B + C mean). Significance level is indicated as *p*_total_.

*p < 0.05;

**p < 0.01;

***p < 0.001;

****p < 0.0001.

**Table 10.** Exploration times and discrimination indexes during the practice phase in the retrieval practice condition for experiment depicted in Figure 3*A*, double practice sessions

	Saline			SKF 38393			*p*_total_	*n*
	A	X/Y/Z	DI	A	X/Y/Z	DI		
RP-2								
S1	12.92 ± 0.50	20.3 ± 1.76	0.21 ± 0.03	14.6 ± 1.84	22.04 ± 2.33	0.21 ± 0.03	0.40	5
S2	10.9 ± 2.15	16.52 ± 3.01	0.23 ± 0.05	10.14 ± 0.87	16.3 ± 1.30	0.23 ± 0.04	0.85	5
	X/Y/Z	X/Y/Z	DI	X/Y/Z	X/Y/Z	DI	*p*_total_	*n*
IC2								
S1	18.36 ± 1.23	19.24 ± 1.00	0.025 ± 0.03	18.76 ± 3.74	21.06 ± 3.88	0.06 ± 0.008	0.78	5
S2	14.8 ± 1.73	16.02 ± 1.93	0.039 ± 0.03	14.4 ± 3.62	16.51 ± 4.04	0.074 ± 0.01	0.99	5

Total exploration times during the retrieval practice phase and DI for the RP-2 and IC2 groups when animals were infused with SKF 38393 or saline in the mPFC. Values are expressed in seconds (mean ± SEM). Paired Student's *t* test, comparing total exploration time between saline- and SKF-injected animals for each retrieval practice session (e.g., saline A + X mean vs SKF A + X mean for RP group and SKF X_1_ + X_2_ mean vs saline X_1_ + X_2_ mean, for IC group). Significance level is indicated as *p*_total_. SKF 38393 injection did not affect total exploration times during the practice phase compared with saline injection.

**Table 11.** Exploration times during the final test phase for experiment depicted in Figure 3*A*, double practice sessions

	Saline				SKF 38393				*p*_total_	*n*
	Object B	Object C	*p*	Total	Object B	Object C	*p*	Total		
RP-2	20.76 ± 2.53	20.58 ± 1.91	NS	41.34 ± 3.99	17.08 ± 1.48	17.86 ± 1.48	NS	34.94 ± 3.05	0.09	5
IC2	16.70 ± 1.72	31.88 ± 2.48	**	48.58 ± 3.30	14.98 ± 1.85	30.10 ± 3.38	**	45.08 ± 5.02	0.48	5
RP + 2	18.34 ± 3.48	27.16 ± 4.72	**	45.5 ± 5.24	12.38 ± 2.72	24.82 ± 5.95	***	37.2 ± 7.67	0.24	5

Absolute exploration time during the final test phase. Total exploration times during the final test phase for the RP-2, IC2, and TC conditions. Values are expressed in seconds (mean ± SEM). Paired Student's *t* test, comparing individual object exploration time between saline- and SKF-injected animals for the test phase; significance level is indicated as *p*. Paired Student's *t* test, comparing total exploration time between saline- and SKF-injected animals for the test phase (e.g., SKF 38393 B + C mean vs saline B + C mean). Significance level is indicated as *p*_total_.

*p < 0.05;

**p < 0.01;

***p < 0.001;

****p < 0.0001.

We found an alternative approach to potentially observe a positive modulation of retrieval-induced forgetting. We introduced a longer delay in between the encoding phase, the practice phase, and the final test, a manipulation that significantly reduced the size of retrieval-induced forgetting. We extended the delay between the encoding and final test phase to 48 h, and the delay between the encoding and the retrieval practice phases to 24 h (Fig. 3*C*, scheme) with the aim of weakening the overall effect so that positive modulation could be observed. To ensure that memory performance was adequate to measure retrieval-induced forgetting after 48 h, we modified our encoding protocol to create stronger memories. Preliminary work indicated that control animals required two separate exposures to each pair of objects during encoding to remember these objects 48 h later. Thus, we slightly modified the protocol for the particular mnemonic demands of longer-lasting object memories.

Using this protocol, the discrimination index in the RP group injected with Veh was not significantly different from that of the IC or TC groups after three practice sessions (Fig. 3*D*, Table 12; one-way ANOVA; Condition: *p* = 0.212, *F*_(1.98,35.7)_ = 4.055; Animals: *p* = 0.36, *F*_(18,54)_ = 1.12; multiple comparisons). Critically, however, injection of the D_1_R agonist SKF into mPFC 10 min before the beginning of the retrieval practice session produced a robust reduction in the discrimination index in the RP condition compared with the control groups (Fig. 3*F*, Tables 13, 14; two-way ANOVA; Interaction: *p* < 0.0001, *F*_(2,23)_ = 25.50; Drug: *p* = 0.0016, *F*_(1,23)_ = 12.85; Condition: *p* = 0.013, *F*_(23,23)_ = 3.41; Bonferroni's *post hoc* multiple-comparisons test). These findings are consistent with the possibility that SKF amplified the capacity of the mPFC to hinder competing memories, enabling retrieval-induced forgetting even after 48 h.

**Table 12.** Exploration times and discrimination indexes during final test for experiment depicted in Figure 3*C*, normal practice phase

	Object B	Object C	*p*	DI	Total	*n*
RP–	19.68 ± 2.08	37.78 ± 3.62	**	0.30 ± 0.06	57.46 ± 4.22	19
IC	16.87 ± 1.42	35.98 ± 2.53	**	0.35 ± 0.04	52.85 ± 3.22	19
TC	14.81 ± 1.12	37.69 ± 2.11	***	0.44 ± 0.02	52.5 ± 2.88	19
RP+	13.08 ± 1.26	37.03 ± 2.56	***	0.49 ± 0.03	50.11 ± 3.47	19

Absolute exploration time during the final test. Total exploration scores during the test phase for the RP-, IC, TC, and RP+ conditions. Values are expressed in seconds (mean ± SE). Within-subject experiment. Paired Student's *t* test, comparing individual object exploration time for the test phase; significance level is indicated as *p*.

*p < 0.05;

**p < 0.01;

***p < 0.001;

****p < 0.0001.

**Table 13.** Retrieval practice exploration times and discrimination indexes for experiment depicted in Figure 3*F*, normal practice phase with SKF 38393 infusion into the mPFC

	Saline			SKF 38393			*p*_total_	*n*
	A	X/Y/Z	DI	A	X/Y/Z	DI		
RP								
S1	16.41 ± 2.95	24.76 ± 2.44	0.21 ± 0.07	15.8 ± 1.34	28.01 ± 3.89	0.23 ± 0.07	0.74	8
S2	8.9 ± 0.89	18.41 ± 4.32	0.35 ± 0.08	13.71 ± 2.87	18.18 ± 2.54	0.17 ± 0.08	0.50	8
S3	10.86 ± 1.59	23.23 ± 5.49	0.40 ± 0.06	7.45 ± 1.04	23.39 ± 3.50	0.48 ± 0.07	0.68	8
	X/Y/Z	X/Y/Z	DI	X/Y/Z	X/Y/Z	DI	*p*_total_	*n*
IC								
S1	18.72 ± 2.78	19.87 ± 2.45	0.05 ± 0.05	16.38 ± 2.22	16.98 ± 1.58	0.03 ± 0.04	0.42	8
S2	15.22 ± 2.47	16.68 ± 2.34	0.06 ± 0.03	15.39 ± 2.22	17.32 ± 1.94	0.07 ± 0.05	0.88	8
S3	17.99 ± 4.77	18.52 ± 4.83	−0.00 ± 0.04	15.31 ± 3.17	14.74 ± 2.87	−0.03 ± 0.03	0.58	8

Absolute exploration time during the retrieval practice phase. Total exploration times during the retrieval practice phase and DI for the RP and IC groups when animals were infused with SKF 38393 or saline in the mPFC. Values are expressed in seconds (mean ± SEM). Paired Student's *t* test, comparing total exploration time between saline- and SKF-injected animals for each retrieval practice session (e.g., saline A + X mean vs SKF A + X mean for RP group and saline X_1_ + X_2_ mean versus SKF X_1_ + X_2_ mean, for IC group). Significance level is indicated as *p*_total_. SKF 38393 injection did not affect total exploration times during the practice phase compared with saline injection.

**Table 14.** Exploration times during the final test phase for experiment depicted in Figure 3*C*, normal practice phase with SKF 38393 infusion into the mPFC

	Saline				SKF 38393				*p*_total_	*n*
	Object B	Object C	*p*	Total	Object B	Object C	*p*	Total		
RP–	11.84 ± 0.88	34.04 ± 3.99	***	45.88 ± 4.31	18.96 ± 2.31	22.95 ± 3.85	0.18	41.91 ± 5.76	0.47	8
IC	13.34 ± 1.58	34.02 ± 4.64	***	47.37 ± 5.99	10.70 ± 1.36	34.03 ± 5.41	**	44.73 ± 6.35	0.69	8
TC	11.66 ± 1.65	33.60 ± 5.04	***	45.26 ± 6.11	11.43 ± 1447	35.93 ± 7.28	**	47.37 ± 8.16	0.81	9
RP+	12.93 ± 1.47	33.58 ± 3.95	**	48.01 ± 3.90	11.27 ± 1054	36.74 ± 4.23	***	46.51 ± 4.86	0.75	8

Absolute exploration time during the final test phase. Total exploration times during the final test phase for the RP–, IC, TC, and RP+ conditions. Values are expressed in seconds (mean ± SEM). Paired Student's *t* test, comparing individual object exploration time between saline- and SKF-injected animals for the test phase; significance level is indicated as *p*. Paired Student's *t* test, comparing total exploration time between saline- and SKF-injected animals for the test phase (e.g., SKF 38393 B + C mean versus saline B + C mean). Significance level is indicated as *p*_total_.

*p < 0.05;

**p < 0.01;

***p < 0.001;

****p < 0.0001.

To confirm that retrieval-induced forgetting could also occur in this longer protocol, we added two extra retrieval practice sessions to the practice phase (Fig. 3*C*, scheme, extended practice). In Veh-infused rats, five retrieval practice trials induced significant reductions in the discrimination index during the final test for the RP condition, even at the 48 h postencoding delay compared with matched IC and TC control groups (Fig. 3*E*, Table 15; one-way ANOVA; Condition: *p* = 0.0002, *F*_(1.98,13.89)_ = 17.47; Animals: *p* = 0.092, *F*_(7,14)_ = 2.25; multiple comparisons). Injection of the D_1_R antagonist SCH into mPFC 10 min before the first of the five practice trials completely prevented this reduction in the discrimination index on the final test of RP items, as performance was indistinguishable from that of the IC and TC groups (Fig. 3*G*, Tables 16, 17; two-way ANOVA; Interaction: *p* < 0.038, *F*_(3,30)_ = 3.18; Drug: *p* = 0.0009, *F*_(1,30)_ = 13.48; Condition: *p* < 0.0001, *F*_(3,30)_ = 11.51; Bonferroni's *post hoc* multiple-comparisons test). Together, these findings are consistent with a bidirectional modulation of retrieval-induced forgetting by manipulation of dopaminergic signaling through D_1_Rs in the mPFC.

**Table 15.** Exploration times and discrimination indexes during final test for experiment depicted in Figure 3*C*, extended practice phase

	Object B	Object C	*p*	DI	Total	*n*
RP	21.8 ± 1.79	24.39 ± 2.02	0.34	0.05 ± 0.04	46.19 ± 2.85	7
IC	13.17 ± 1.63	26.3 ± 2.89	**	0.44 ± 0.03	43.34 ± 3.86	7
TC	13.97 ± 1.62	29.37 ± 2.52	***	0.31 ± 0.05	59.29 ± 4.73	7
RP+	16.03 ± 1.01	43.26 ± 4.02	***	0.35 ± 0.03	39.47 ± 3.86	7

Absolute exploration time during the final test phase. Total exploration scores during the test phase for the RP–, IC, TC, and RP+ conditions. Values are expressed in seconds (mean ± SE). Within-subject experiment. Paired Student's *t* test, comparing individual object exploration time for the test phase; significance level is indicated as *p*.

*p < 0.05;

**p < 0.01;

***p < 0.001;

****p < 0.0001.

**Table 16.** Retrieval practice exploration times for experiment depicted in Figure 3*G*, extended practice phase with SCH 23389 infusion into the mPFC

	Saline			SCH 23389			*p*_total_	*n*
	A	X/Y/Z	DI	A	X/Y/Z	DI		
RP								
S1	15.39 ± 1.64	26.68 ± 2.51	0.26 ± 0.06	12.14 ± 1.58	26.82 ± 2.43	0.29 ± 0.05	0.10	9
S2	14.36 ± 1.57	29.18 ± 3.14	0.33 ± 0.04	10.66 ± 1.34	24.07 ± 2.65	0.37 ± 0.04	0.12	9
S3	11.89 ± 1.72	23.24 ± 2.03	0.28 ± 0.07	8.63 ± 1.18	23.16 ± 2.45	0.268 ± 0.05	0.39	9
S4	11.14 ± 1.91	24.46 ± 4.33	0.31 ± 0.09	9.08 ± 1.72	21.81 ± 2.33	0.32 ± 0.06	0.30	9
S5	10.02 ± 1.40	25.16 ± 3.48	0.41 ± 0.04	7.96 ± 2.25	16.68 ± 2.41	0.45 ± 0.08	0.21	9
	X/Y/Z	X/Y/Z	DI	X/Y/Z	X/Y/Z	DI	*p*_total_	*n*
IC								
S1	19.71 ± 3.05	21.45 ± 2.04	0.07 ± 0.06	16.24 ± 2.90	18.89 ± 2.05	0.11 ± 0.04	0.57	7
S2	19.41 ± 1.88	19.06 ± 1.39	0.02 ± 0.01	17.09 ± 1.63	18.15 ± 2.49	0.03 ± 0.04	0.75	7
S3	15.79 ± 1.74	15.03 ± 2.15	−0.03 ± 0.02	9.57 ± 2.32	13.01 ± 1.65	0.05 ± 0.12	0.27	7
S4	14.39 ± 2.77	15.14 ± 3.10	−0.02 ± 0.04	9.39 ± 1.43	10.69 ± 3.03	−0.01 ± 0.08	0.14	7
S5	12.89 ± 2.83	11.73 ± 2.83	−0.00 ± 0.06	10.81 ± 2.71	13.21 ± 2.49	0.10 ± 0.09	0.98	7

Absolute exploration time and discrimination indexes during the retrieval practice phase for experiment 10. Total exploration times during the retrieval practice phase and DI for the RP and IC groups when animals were infused with SCH 23389 or saline in the mPFC. Values are expressed in seconds (mean ± SEM). Paired Student's *t* test, comparing total exploration time between saline- and SCH-injected animals for each retrieval practice session (e.g., saline A + X mean vs SCH A + X mean for RP group and saline X1 + X2 mean versus SCH X1 + X2 mean, for IC group). Significance level is indicated as *p*_total_. SCH 23389 injection did not affect total exploration times during the practice phase compared with saline injection.

**Table 17.** Exploration times during the final test phase for experiment depicted in Figure 3*C*, extended practice phase with SCH 23389 infusion into the mPFC

	Saline				SCH 23389				*p*_total_	*n*
	Object B	Object C	*p*	Total	Object B	Object C	p	Total		
RP–	18.72 ± 1.98	21.38 ± 1.85	0.0661	40.1 ± 3.62	14.36 ± 1.12	30.7 ± 3.50	**	45.06 ± 3.87	0.32	9
IC	14.47 ± 0.91	29.19 ± 4.57	*	43.66 ± 5.03	11.57 ± 1.30	23.9 ± 2.58	***	35.47 ± 3.73	0.25	7
TC	13.38 ± 1.18	28.41 ± 2.86	***	41.79 ± 3.64	12.63 ± 1.07	30.22 ± 2.81	***	42.86 ± 3.34	0.83	9
RP+	13.00 ± 1.16	31.07 ± 2.02	****	44.07 ± 2.8	10.8 ± 1.57	31.58 ± 4.05	***	42.38 ± 5.09	0.76	9

Absolute exploration time during the final test phase. Total exploration times during the final test phase for the RP-, IC, TC, and RP+ conditions. Values are expressed in seconds (mean ± SEM). Paired Student's *t* test, comparing individual object exploration time between saline- and SCH-injected animals for the test phase; significance level is indicated as *p*. Paired Student's *t* test, comparing total exploration time between saline- and SCH-injected animals for the test phase (e.g., SCH 23389 B + C mean vs saline B + C mean). Significance level is indicated as *p*_total_.

*p < 0.05;

**p < 0.01;

***p < 0.001;

****p < 0.0001.

## Discussion

Memory enables organisms to draw on past experiences to improve their choices and actions. Because of their relational nature and richness, episodic memories are flexible in the way that past events can be retrieved as needed to guide future behavior (Eichenbaum and Cohen, 2001). Experience modifies behavior by restructuring access to memories or directly modifying the memory traces themselves (Quirk and Mueller, 2008; Lee, 2009; Medina, 2018). Dopamine plays important functions in the ability to change a learned rule and to select appropriate behaviors (Seamans and Yang, 2004) by biasing action selection and even by modifying neural plasticity in regions of memory storage (Lisman and Grace, 2005; Neugebauer et al., 2009). In this work, we expand the functions of dopamine to include a mechanism of adaptive forgetting of competing memories. Although the role of dopamine has been studied mainly in the motivation of goal-directed behaviors, here we argue that dopamine-dependent mechanisms are related to adaptive forgetting even in the absence of explicit reward or instructions. We propose that retrieval-induced forgetting of competing object memories is enabled by mechanisms similar to those engaged during rule switching and selection in the mPFC of rodents. This dopaminergic modulation of control processes enables access to memory content in the face of retrieval competition, supporting the behavioral demands of organisms.

Remarkably, retrieval-induced forgetting in rats resembles the corresponding process in humans (Bekinschtein et al., 2018). The mPFC in rats is essential to forget competing object memories, paralleling results observed for the lateral prefrontal cortex in humans. These results point to the key role of inhibitory control in retrieval-induced forgetting. We provide strong causal evidence favoring a dopamine-dependent mechanism of inhibitory control for retrieval-induced forgetting. Blockade of D_1_Rs in the mPFC of rats during the practice phase abolished retrieval-induced forgetting of a competing object memory. This manipulation did not have any effect when it preceded the encoding of different interfering materials (interference control) or when it preceded rest in the home cage of rats, indicating that it affected processes specifically associated with retrieval practice and not nonspecific factors such as novelty salience or mood, which would have affected performance in all three conditions. The function of D_1_Rs in the prefrontal cortex has been extensively investigated. D_1_R blockade in nonhuman primates disrupts task performance and spatial working memory activity in the dorsolateral prefrontal cortex (Sawaguchi and Goldman-Rakic, 1991, 1994; Williams and Goldman-Rakic, 1995). Importantly, D_1_R blockade also disrupts prefrontal cognitive rule-related selectivity (Ott et al., 2014). In this work, we found that the same dose and place of infusion of the D_1_R antagonist that prevented retrieval-induced forgetting also impaired performance in a set-shifting task in which rats are required to inhibit a prepotent response associated with a learned rule. The parallel impact of a D_1_R antagonist on the need to inhibit prepotent actions and memories is consistent with human studies indicating that retrieval-induced forgetting is triggered by inhibitory control processes shared with action stopping (Schilling et al., 2014; Anderson and Hulbert, 2021; Apšvalka et al., 2022). It also provides new evidence in favor of a general function of dopamine in cognitive processes related to flexible and adaptive behavior.

We provided causal evidence that the critical source of dopamine for retrieval-induced forgetting in the mPFC is the VTA, because silencing this structure impaired retrieval-induced forgetting. This effect was reversed by concomitant activation of D_1_Rs in mPFC during the practice phase, indicating that, in the absence of dopamine release from VTA, the activation of D_1_Rs in the mPFC is sufficient for retrieval-induced forgetting. Critically, dopaminergic modulation of retrieval-induced forgetting is bidirectional. Activation of D_1_Rs in the mPFC just before the retrieval practice phase caused retrieval-induced forgetting in a protocol that does not reliably induce it without D_1_R activation. No anxiety, movement, or perception changes were observed after any of the infusions, as rats did not significantly modify their exploratory behavior after the infusion of any of the drugs.

There is a strong link between dopamine availability in the brain and cognitive abilities. Many studies point at a function of dopamine in adaptive behavior in humans. For example, the administration of l-DOPA to Parkinson's disease patients improved the ability to alter behavior according to changes in the dimensional relevance of stimuli in a task that resembles the set-shifting paradigm used in our study (Cools et al., 2001). Impairments in this form of higher-level attentional control have also been associated with lesions of the monkey lateral PFC (Dias et al., 1996) and significant activation of the dorsolateral prefrontal cortex in humans (Rogers et al., 2000; Nagahama et al., 2001). In addition, the enzyme COMT, which degrades catecholamines, appears to play a pivotal role in the modulation of frontostriatal networks. The *COMT* gene presents an evolutionarily recent functional single nucleotide polymorphism (Val158Met). The Met allele produces an enzyme that has only a quarter the activity of the Val-containing polypeptide (Egan et al., 2001). Several studies found that the low-activity Met allele allows for better performance on cognitive tasks that have a working memory component and the high-activity Val allele was associated with poorer performance on the Wisconsin Card Sorting Test, a putative measure of “executive” function (for review, see Savitz et al., 2006). Interestingly, in humans, retrieval-induced forgetting increased linearly with Met allele load, suggesting a positive relationship between cortical dopamine availability and inhibitory control over memory (Wimber et al., 2011). Mirroring the linear effect of genotype on behavior, functional imaging data revealed that the beneficial effects of memory suppression, as assessed by a decrease in prefrontal activity across retrieval practice blocks, a sign of efficient suppression of competing memories (Kuhl et al., 2007; Bekinschtein et al., 2018; Anderson and Hulbert, 2021), also increased with Met allele load. In agreement with these results, the present study supports a general contribution of dopamine in the mPFC in the control of memory and, in particular, establishes causality between dopamine availability and retrieval-induced forgetting. Greater dopamine availability may lead to greater activation of D_1_Rs, improving the suppression of competing memories.

What are the mechanisms by which dopamine participates in retrieval-induced forgetting? Activation of D_1_Rs in mPFC could initiate active circuit-level inhibition over competing memory traces in the medial temporal lobe. Given that top-down connections from the mPFC to the medial temporal lobe are mainly excitatory (Hoover and Vertes, 2007; Vertes et al., 2007) projections from the mPFC would not directly enact inhibition over the competing memory trace. A possible mechanism could involve excitatory projections from the prefrontal cortex that directly excite local inhibitory neurons in the medial temporal lobe, which then inhibit a distracting stimulus, or unwanted representation or process (Chamberland and Topolnik, 2012), but this remains highly speculative. Since there are no direct projections from the mPFC to the hippocampus, the activation of the mPFC could induce inhibition of the competing traces in the hippocampus via nucleus reuniens (RE; Anderson et al., 2016). Anderson and Floresco (2022) also developed this mPFC-RE-Hippocampus model in the context of memory inhibition during extinction.

Regardless of the circuit involved in retrieval-induced forgetting, we made the surprising discovery that dopaminergic modulation of retrieval-induced forgetting seems to be independent of any mechanisms of retrieval itself (i.e., D_1_R blockade in mPFC does not affect retrieval during the practice phase but impairs retrieval-induced forgetting). This suggests that dopamine modulates retrieval-induced forgetting by specifically acting on the future availability of the competing memory trace (i.e., at the test phase), without affecting the retrieval processes during the practice phase. Thus, we argue that retrieval control and retrieval-induced forgetting mechanisms are intrinsically distinct. During retrieval practice, activity in the mPFC would be required for inhibition of the competing memory, but not for the mechanism of retrieval itself. Lesions to the mPFC in rats do not normally impair object recognition when the task relies on the identity of the object (Warburton and Brown, 2015). However, what we found is that even if the mPFC is not implicated in object memory retrieval, it does not mean that the structure does not participate in memory retrieval at all. In particular, D_1_Rs would be essential for high-level function of the mPFC. As would be expected given the plethora of diffuse ascending inputs from the major monoaminergic and cholinergic neurotransmitter systems, the PFC needs to be highly sensitive to neurochemical state. In particular, in set-shifting tasks, the modulation of noradrenaline usually produces similar effects to the modulation of the dopaminergic system (McGaughy et al., 2008; Tait et al., 2014). Thus, it is possible that noradrenaline is also involved in retrieval-induced forgetting. However, we found that the activation of D_1_Rs after silencing the VTA restored retrieval-induced forgetting, indicating that dopaminergic release may be a key step for this process. It is clear that acetylcholine and serotonin can also modulate mPFC activity, but their manipulation does not seem to produce the same behavioral effects as that of dopamine or noradrenaline in tasks that involve attention and control processes, although there is some complex interaction between dopamine and serotonin to modulate PFC function (Boulougouris and Tsaltas, 2008; Tait et al., 2014). So, the neurochemical processes involved in retrieval-induced forgetting require a thorough evaluation.

Two limitations of our study could be addressed in the future. First, for simplicity, we only analyzed the effect of a single dose of each ligand. Because dopamine exerts a complex modulation of cortical function (Robbins, 2005; Floresco et al., 2006; Floresco, 2013) in future studies, it will be helpful to analyze the effects of other doses to examine whether the modulation of retrieval-induced forgetting follows the same pattern as has been observed in other cognitive functions. Second, we only studied male subjects. Our current work uses both sexes, which will generalize the conclusions that we might obtain. The present study is just one of the first steps toward understanding the biological mechanisms underlying retrieval-induced forgetting.

In agreement with an adaptive and evolutionarily conserved role in memory and behavior, dopamine has been recently implicated in forgetting mechanisms in both invertebrates (Berry et al., 2012) and vertebrates (Wimber et al., 2011; Castillo Díaz et al., 2019). Modulation of a small subset of dopaminergic neurons in *Drosophila* regulates the rate of forgetting of aversive and rewarding experiences. In particular, forgetting appears to depend on signaling through a specific type of receptor in the mushroom bodies of the fly brain (Berry et al., 2012). On the other hand, inhibition of D_1_Rs in the VTA during training of a conditioned place preference task in rats, increases memory duration, while activation of these receptors produces forgetting of already consolidated memories (Castillo Díaz et al., 2019). In the absence of any type of retrieval practice, blockade of mPFC D_1_Rs did not produce forgetting of the conditioned place preference memory. Although they did not evaluate the function of D_1_Rs in retrieval-induced forgetting, it does contribute to an increasing accumulation of evidence for the involvement of the dopaminergic system in the different mechanisms of forgetting linked to adaptive behavior.

According to our results, dopamine acting on D_1_Rs in the mPFC modulates control processes required for adaptive forgetting in the mammalian brain. Thus, across species, dopaminergic transmission may be essential to suppress competing memories by sculpting the mnemonic and behavioral repertoire of an organism according to their goals and the demands of the environment.
